# Supplementary material for: Complexome profiling of the Chlamydomonas psb28 mutant reveals TEF5 as an early PSII assembly factor
Source: Plant Cell. 2025 Mar 17;37(6):koaf055. doi: 10.1093/plcell/koaf055 (PMC12164590; doi:10.1093/plcell/koaf055)
Supplement: koaf055_Supplementary_Data [file koaf055_supplementary_data.zip › psb28_interactive.zip!/plots/intensity/qualityPlots/peptides/nad4_Cre-2.g801494.t1.p1.html]

### Cre-2.g801494.t1.p1

Trivial name: nad4  
SeqCoverage [median]: 7.40  
Mass: 48.74 kDa  
  
 

| Sequence | MissedCleavage | Mass [Da] | wtIntensity | muIntensity | Score |
| --- | --- | --- | --- | --- | --- |
| APYGSLEAAYK | 0 | 1168.58 | 2122.99 | 1156.53 | 107.15 |
| GPADVTR | 0 | 714.37 | 13308.51 | 19318.41 | 148.33 |
| LGGIGFLR | 0 | 831.50 | 9374.38 | 0.00 | 114.54 |
| VVHGLPR | 0 | 776.47 | 5270.90 | 5138.53 | 91.09 |
|  |  |  | 30076.8 | 25613.5 |  |
